# Spinal Curvature

**Published:** 1903-05

**Authors:** Theo. Toepel

**Affiliations:** Atlanta, Ga.


### SPINAL CURVATURE.

By THEO. TOEPEL, M.D.,

ATLANTA, GA.

It would be impossible for me to do the subject spinal curvature justice in a brief way. I have therefore selected that portion which I think will be of interest, and which occurs most frequently, namely: the acquired form of lateral spinal curvature.

In this seemingly small portion of the main subject it is not my idea to cover all the details, but shall only point out a few conditions that are most commonly met with.

To define lateral curvature briefly, I would say it is a deformity of the spine, which is characterized by lateral deviation and distortion or rotation of the spinal column, nearly always accompanied by a more or less exaggeration or diminution of the antero-posterior curves.

The causes of acquired lateral curvature are manifold—some of the most important, and those most frequently met with, are asymmetry of the head, asymmetry of the pelvis, short leg, and asymmetry of the spine itself. Asymmetry of the head is a factor not often taken into account. Ocular defects, causing improper balance of the head, are an obvious cause. Asymmetry of the pelvis, as described by Barwell, is accounted as a not infrequent cause of asymmetrical positions. The shortness of one leg is so common that it fails to attract the attention that it deserves. Asymmetry of the spine itself, and unequal thickness of the vertebral bodies on the right side, as contrasted with the left, is a demonstrated condition.

All these defects of conformation must lead to some lateral deviation of the spine. It depends upon the individual whether these are taken care of by nature in some compensatory way, or whether they cause a deviation notable enough to be classed as deformities.

Aside from the structural defects, causing asymmetry and consequent lateral deviation, are to be found the faulty postures resulting from attitude and occupation, which must be accounted another important factor in producing lateral curvature. Habitual lateral bed postures curve the spine. By favoring the growth of the spine on one side, and retarding it on the other, there is a tendency to fix a lateral curve by establishing physical changes in the bones and ligaments.

The twisted position in writing, as generally practiced, is more frequently than anything else an initial cause of many lateral curvatures. When examining for lateral curvature, after having noted the kind and degree of curvature, let the patient sit down and write his or her name. Nine times out of ten the patient will have placed himself in a posture corresponding with the form of the curvature, except that usually it is highly exaggerated. Playing the violin in the usual standing position has no doubt contributed to bring on lateral curvature. Other similar causes might be enumerated, but the aforesaid will suffice to look for these and related conditions in examination.

The usual history given is that the mother or dressmaker has noticed one shoulder-blade or hip bone growing out. Then at a convenient time the family physician is called in. Generally he does not strip the patient below the hip bones, and then tell him to bend the trunk forward, or often he only passes his hand down the back over the clothing. If there is no decided or marked irregularity of the back, when examined in this haphazard way, the doctor often assures the anxious mother that the patient will outgrow it, and that nothing special need be done, except perhaps to lie down for two or three hours daily, or to use dumb-bells or calisthenic exercise.

Pain and backache, generally of the loins or under or in the neighborhood of one shoulder-blade, or between the shoulder-blades, are the first symptoms observed in a large number of cases. This is frequently attributed to something entirely different, and treated accordingly.

The prognosis in most cases is favorable, if presented in the early stage, and when there is not osseous deformity present, such patients can be assured complete cure. If osseous deformity is present the case is incurable. Before lateral curvature with osseous deformity can occur, it must gradually pass through many intermediate stages from the time the patient first began to assume a temporary faulty position of the trunk.

Most surgeons are now agreed that in lateral curvature osseous deformity is preceded by so-called postural deformity. As soon as osseous deformity is present not only do the individual vertebræ become wedge-shaped and misshapen with the attached ribs, but the actual bony fibers of the spongy tissue of the bodies of the vertebræ participate in the deformation, and show in sections from above downward a twisting corresponding to the rotation deformity of the whole adjacent group of vertabræ.

When accepting a case of lateral curvature for treatment I make it a rule to note the lateral deviation carefully, and at regular intervals make comparative notations of the improvement. A strip of adhesive plaster two inches wide is placed over the vertebral column. With a colored pencil the position of each successive spine is marked on the adhesive plaster. I also mark the level of the tips of the scapulæ on the corresponding margins of the plaster, also the transverse line between the posterior superior spines of the ilium is drawn to record any possible lateral tilt of the pelvis. The adhesive strip is now removed to a book where these records are kept. With a straight-edged ruler a line is drawn between the position of the seventh cervical and first sacrum spine. The amount of deviation in centimeters is easily measured. It corresponds to the distance from this line to the farthest removed dot on either side.

Of late the implicit faith formerly placed in the treatment of lateral curvature of the spine by steel and other spinal supports or stays has been gradually undermined, and even those who still adhere to the mechanical treatment of spinal deformities not due to diseased bone attach increasing importance to its association with suitably prescribed gymnastics.

The treatment is based upon principles which may be taken under the following heads:

(a) Re-education of the patient's muscular sense as to an erect and improved position. This re-education of the muscular sense for the improved and normal posture is to be kept in mind throughout the whole treatment.

(b) Improved position to be always maintained while sitting, standing or lying. The best possible posture is to be maintained while sitting, whatever the occupation of the moment may be. It is most readily obtained by sitting with the sacrum, loins, dorsum and shoulders well supported against the back of the chair, which should be molded to the normal shape of the spine. Standing at lessons, or any other occupation, should be avoided when possible; when unavoidable the patient ought to stand on both feet, with heels three to four inches apart. Standing on one foot is most injurious, as it at once throws the spine into a serpentine position. A short leg may be compensated by a thicker sole. As the time spent in sleep is from one third to one half during the growing period, a proper bed posture is an important factor in the treatment.

(c) Attention to dress. It is essential that no article of clothing should interfere with the resumption of an improved and perfectly normal position of the patient's spine and trunk.

(d) Systematic training of the spinal and other muscles, including the development of the thorax. The exercises are divided into three distinct sets, varying according to the patient's requirements, namely: 1. Exercises where the patient lies on his back or abdomen, extension from the sacrum upward, or suspended from rings, bars, or Dr. Schmidt's spinal stretcher, extension from neck downward. 2. Exercises in sitting. 3. Exercises in standing.

In case of non-osseous or postural lateral curvature five to six months' daily treatment will effect a cure of the deformity, while in cases with osseous or incurable deformity one year's daily treatment will effect all the improvement that is possible. Very severe cases, especially those associated with much pain, require a longer period of treatment. The great advantage of this treatment of lateral curvature over that by spinal supports is that it always tends to improve the general health of the patient, notably in delicate anemic and poorly nourished boys and girls at the onset of puberty.

(e) Attention to general health. Care should be taken to improve the general health in every possible way. A nutritious diet should be prescribed. A daily bath with cold or tepid water, if the patient's powers of reaction are low, should always be insisted upon as a general tonic. At least one and a half hours to two daily outdoor exercise should be prescribed. Very frequently patients gain at the rate of half a pound to a pound every week.

(f) Subsequent home treatment to prevent a relapse in the improvement or cure that has been accomplished. To keep up the improvement, and to prevent relapse in a cured case, it is necessary to give the patient, on leaving, a written home prescription of exercises.

